# The critical role of timely medical emergency team activation in oncological and non-oncological patients

**DOI:** 10.1371/journal.pone.0324831

**Published:** 2025-06-02

**Authors:** Kaspar F. Bachmann, Samuel J. Michimura, Luca Cioccari, Joerg C. Schefold, Anna S. Messmer

**Affiliations:** 1 Department of Intensive Care Medicine, Inselspital, Bern University Hospital, University of Bern, Bern, Switzerland; 2 Department of Intensive Care Medicine, Kantonsspital Aarau, Aarau, Switzerland; Universitas Airlangga, INDONESIA

## Abstract

**Purpose:**

This study aimed to analyse the impact of time from first physiological derangement (positive Vital Sign Score, VSS) to Medical Emergency Team (MET) activation on mortality in oncological versus non-oncological patients.

**Methods:**

All in-hospital patients requiring a MET assessment were included. The primary outcome was 30-day mortality. Subanalyses were performed for the population that was admitted to the ICU as well as the subgroups of oncological with solid vs. haematological cancer. In addition, we assessed the number and time points of VSS measurements in oncological and non-oncological patients.

**Results:**

286 out of 4068 (8.9%) documented MET calls were attributed to oncological patients. Each hour delay from first abnormal VSS to MET activation was associated with an 3% increase in adjusted 30-day all-cause mortality odds (OR 1.03, 95% CI 1.02–1.05, p < 0.001), independent of oncological status (interaction p = 0.141). Oncological patients had a significantly higher number of vital sign measurements on the ward (median 24 [16–37] vs 18 [10–27], p < 0.001) before MET activation, and higher 30-day all-cause mortality (38.1% vs 21.8%, p < 0.001).

**Conclusions:**

Although the overall outcome did not differ between, oncological patients comprised 10% of MET calls, had higher mortality and had significantly more frequent vital sign monitoring, reflecting greater clinical concern. Tailored monitoring including early MET review has potential to improve outcomes particularly in high-risk oncological patients.

## Background

Cancer remains one of the most common causes of morbidity and mortality around the globe [1], with approximately 40% of all people worldwide being diagnosed with cancer during their lifetime [2]. With advances in cancer treatment, the long-term outcomes of oncological patients have been improved dramatically, creating new options for conditions previously deemed not curable [3,4]. Over the past decades, patient outcomes have also improved in cancer patients admitted to the intensive care unit (ICU) [5]. Furthermore, ICU survivors with cancer show similar quality of life and remission rates as non-ICU cancer patients [6,7]. Moreover, as a result of the new therapies, intensive care physicians are increasingly confronted with cancer patients requiring ICU admission for infectious or toxic therapy-related events [8–11]. For these patients, early intervention is key, as data has shown admission to the ICU in the early course of their critical illness is associated with better survival rates [6,12,13].

In general, most adverse events are preceded by changes in vital signs, which can be used to anticipate clinical deterioration [14–16]. In addition, previous data has shown that the time from physiological derangement to admission to the ICU is associated with increased mortality [17]. Medical emergency teams (METs) have been introduced as critical care response teams in order to assess and intervene early in deteriorating ward patients [18].

To our knowledge, there is only sparse data about the time of first physical derangement to medical emergency team MET interventions in oncology patients. In addition, it is currently not known if there is a difference between cancer and non-cancer patients in regard to delayed MET interventions.

Thus, this study aims to analyse the impact of time between the first signs of deterioration and MET activation on mortality in oncological and non-oncological patients. Moreover, we aim to describe the characteristics and outcomes of oncological patients requiring a medical emergency team compared to non-oncological patients in a large MET database at a tertiary centre.

## Materials and methods

This retrospective cohort study was conducted at University Hospital Bern (Inselspital) in Switzerland, a tertiary academic centre with approximately 900 beds and an interdisciplinary ICU. This study is part of the MET cohort project, and our institution has previously published an analysis based on the same dataset [18]. Ethical approval was obtained from the Canton Bern Ethics Committee (approval no.: 2019–01260), and the study adhered to the amended Declaration of Helsinki [19]. Only the principal investigator of the project (ASM) had access to information that could identify individual participants for the purpose of data collection. Reporting followed the STROBE guidelines [20].

We included all in-hospital patients requiring a Medical Emergency Team (MET) assessment between January 1, 2012, and May 31, 2019. Out-patients and patients with missing data on vital sign measurements were excluded. Only the first call was analysed for patients with multiple MET assessments during the same hospitalization. Oncology patients included in this analysis are those who were hospitalized in the medical oncology ward and receiving treatment for active solid or non-solid malignancies. The control group comprised all other patients requiring MET activation without any oncological diseases, patients with malignancy-related surgical treatment, or patients with oncological comorbidities not primarily linked to their hospitalization.

### Data Collection

We analysed prospectively collected data from all MET calls, recorded in a dedicated database. This included patient demographics (e.g., age, sex), time and date of the MET call, start and end of each MET call, parent ward, disposition (e.g., admission to the ICU or intermediate care (IMC) unit, or continuing treatment in on general ward), MET diagnosis, and outcome. Additionally, data on ICU and hospital length of stay, mortality, vital signs up to 24 hours prior to MET call, and hospital diagnosis were extracted from the electronic records for analysis. Data was accessed from December 2019 until October 2022.

### Definitions

Vital sign score: The Vital Sign Score (VSS) criteria include: “threatened airway,” “respiratory rate <6 or >36/min,” “oxygen saturation <90%,” “heart rate <40 or >140 bpm,” “systolic blood pressure ≤90 mmHg,” “Glasgow Coma Score (GCS) <13,” and “concern criteria.” Each abnormal parameter scores one VSS point, summed to calculate the total VSS at a given time. Data included the total number of measurements, those meeting VSS criteria (indicating physiological derangement), and the timing of the first VSS observation. The MET activation criterion in our institution is a VSS > 0, which is deemed abnormal VSS. This includes the “concern criterion”, whereas the team can be activated for any reason at any time by a concerned member of staff.

MET: At our institution, the Medical Emergency Team (MET) is available 24/7 and comprises a board-certified intensive care physician or an intensive care fellow, along with a certified intensive care nurse. Activation of the MET is directed to the designated physician or nurse carrying the MET phone.

### Aims and outcomes

The aim of this study was to evaluate the association between the time from first physiological derangement as defined by VSS > 0 (see below) to MET activation and mortality in oncological and non-oncological patients. The primary outcome was time from first abnormal VSS to MET activation and 30-day mortality. Subanalyses with the same outcome were performed for patients admitted to the ICU as well as the subgroups of oncological with solid vs. haematological cancer. In addition, we assessed the number and time points of VSS measurements in oncological and non-oncological patients.

## Statistical analysis

Data are presented as median with interquartile ranges for continuous variables and numbers with percentages for categorical variables. For baseline characteristics and comparison between groups, logistic regression models were applied with oncological status as the outcome and the variable of interest as the predictor. A log-rank test assessed the time from a VSS score greater than zero to the MET activation. Logistic regression models were used to evaluate the relationship between oncological status, time to MET activation (TTM), and patient mortality.

Three groups were analysed: 1) the entire population, 2) the patients admitted to ICU, and 3) the oncological subgroup (solid vs haematological tumours). Using logistic regression models, we tested whether an oncological diagnosis and the time to MET activation (hours) was associated with mortality, and adjusted for age, admission type and, in the ICU population, disease severity expressed as APACHE II. We added an interaction term of TTM and positive oncological diagnosis, to identify an additional increase in risk in oncological patients with increasing TTM. This estimate is added to the fixed increase estimated by the TTM term. A stepwise backward elimination approach was used to define a baseline model, eliminating variables with a p-value above 0.1 to correct for disease severity. Age, tumour type, and oncological status, as well as APACHEII scores for patients admitted to the ICU were considered important variables to be associated with mortality that could be used for adjustment. All variables initially entered into the models were retained, due to the limited number of available variables that could be considered for adjustment. TTM and oncological status were then added to this model. We adjusted for disease severity for the ICU population using the APACHE II score. For the oncological subgroup, admission type was excluded, as all oncological patients were medical admission. Cases with missing data were excluded from the analysis. Model performance was assessed using multiple measures: the Area under the Receiver Operating Characteristic Curve (AUC-ROC) with bootstrap replicas (n = 500) for bias correction to evaluate discrimination ability, Nagelkerke’s pseudo R² to assess explained variance, and the Hosmer-Lemeshow test to evaluate model calibration across risk groups. Odds ratios (OR) with 95% confidence intervals (CI) were calculated for each predictor variable.

Statistical comparisons of the number of VSS measurements between oncological and non-oncological patients were performed using the Mann-Whitney U test (Wilcoxon rank-sum test) due to the non-normal distribution of these data.

All statistical analyses were performed using MATLAB (v2024a, Mathworks, Massachusetts, USA). A p-value of < 0.05 was considered statistically significant. A more detailed description of the statistical analysis can be found in the supplemental file.

## Results

### Baseline data and patient characteristics

**Fig 1:**
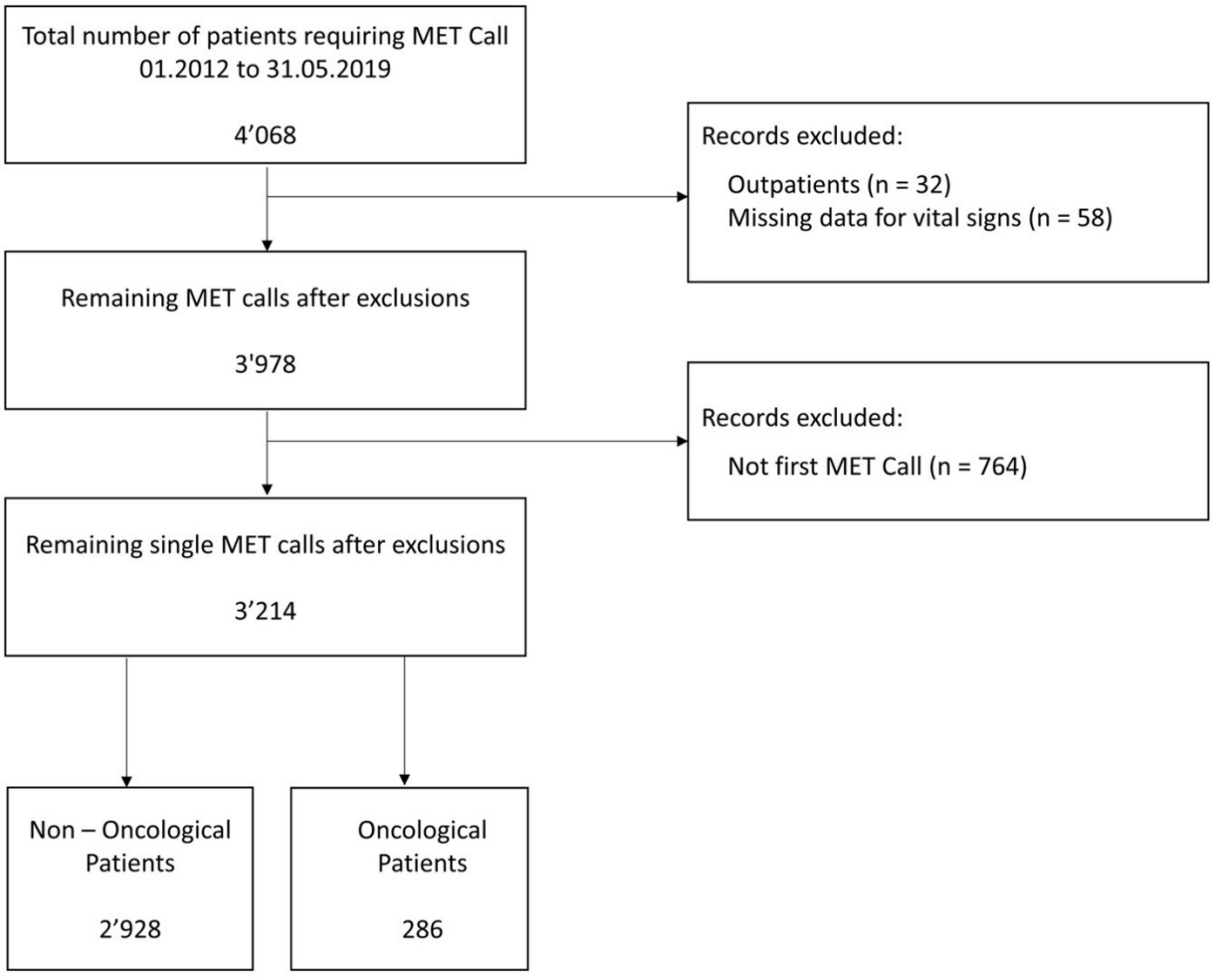
Study flowchart.

The initial database consisted of 4’068 MET calls, of which 396 (9.7%) were attributed to oncological patients. We excluded 32 calls in outpatients, and 58 calls due to missing data. After filtering for the first MET activation only, the dataset was reduced to 3,214 unique calls in 3,214 patients, of which 286 (8.9%) were oncological (Fig 1).

Oncological patients were younger (median age: 62.0 [51.0–69.0] vs 70.0 [58.0–78.0] years, p < 0.001), had a lower BMI (24.7 [21.6–27.9] vs 25.6 [22.3–29.5], p = 0.005), and experienced longer hospital stays (24.0 [12.0–37.0] vs 14.0 [8.0–25.0] days, p < 0.001) compared to non-oncological patients (Table 1). The primary reason for MET activation in both groups was “concern.” (29.6% (951/3214)). Among these, 8.5% (81/951) were oncological patients compared to 91.5% (870/951) non-oncological patients. MET activations triggered by “concern” were associated with lower rates of subsequent ICU/IMC admission compared to other activation criteria (33.6% vs. 56.3%, p < 0.001). Among oncological patients, the most common MET diagnoses were respiratory (21.7%) and infectious (34.3%). Approximately half of the patients in both groups remained on the ward (55.6% oncological vs 49.9% non-oncological), while fewer than half were admitted to the ICU (42.3% oncological vs 45.6% non-oncological). A small proportion was transferred to the intermediate care unit (2.1% oncological vs 4.4% non-oncological, Table 1). Further details on the oncological subgroup are provided in Tables S1 and S2 of the S1 File.

**Table 1:** Baseline characteristics.

Variable	Non-oncological Patients	Oncological	p-value
Age, median years [IQR]	70.0 [58.0-78.0]	62.0 [51.0-69.0]	< 0.001
Sex, N (%)			
- Male	1,848 (63.1)	186 (65.0)	0.52
- Female	1,080 (36.9)	100 (35.0)	
BMI (kg/m2), median [IQR]	25.6 [22.3-29.5]	24.7 [21.6-27.9]	0.005
Admission Type, N (%)			
- Surgical	1,238 (42.3)	0 (0.0)	N/A
- Medical	1,689 (57.7)	286 (100.0)	
MET calling criterion, N (%)			< 0.001
- Systolic blood pressure	414 (16.4)	54 (23.5)	
- Drop in GCS	258 (10.2)	20 (8.7)	
- Concern	870 (34.5)	81 (35.2)	
- Low oxygen Saturation	569 (22.6)	28 (12.2)	
- High or low heart rate	177 (7.0)	29 (12.6)	
- Threatened airway	138 (5.5)	10 (4.3)	
- Seizure	67 (2.7)	3 (1.3)	
- High or low respiratory rate	26 (1.0)	5 (2.2)	
MET diagnosis group, N (%)			
- Neurological	489 (16.7)	24 (8.4)	< 0.001
- Infection - *all*	449 (15.3)	98 (34.3)	
- Infection – *central nervous system*	15 (0.5)	2 (0.7)	
- Respiratory	690 (23.6)	62 (21.7)	
- Cardiovascular	501 (17.1)	26 (9.1)	
- Shock - *haemorrhagic*	89 (3.0)	5 (1.7)	
- Shock - *cardiovascular*	67 (2.3)	1 (0.3)	
- Shock - *septic*	3 (0.1)	0 (0.0)	
- Metabolic, Electrolyte Disturbance	79 (2.7)	2 (0.7)	
- Psychiatric	68 (2.3)	3 (1.0)	
- Gastrointestinal	125 (4.3)	11 (3.8)	
- Renal	15 (0.5)	4 (1.4)	
- Haematological	11 (0.4)	14 (4.9)	
- Trauma	14 (0.5)	0 (0.0)	
- Allergic	3 (0.1)	0 (0.0)	
- Other	310 (10.6)	34 (11.9)	
Disposition, N (%)			
- Intensive care unit	1,336 (45.6)	121 (42.3)	0.065
- IMC	130 (4.4)	6 (2.1)	
- Remain on ward	1,462 (49.9)	159 (55.6)	
Hospital LOS, median days [IQR]	14.0 [8.0-25.0]	24.0 [12.0-37.0]	< 0.001
30- day mortality	637 (21.8)	109 (38.1)	< 0.001

Legend: P-values were calculated using logistic regression, with the variable of interest as predictor and oncological status as the predicted variable. BMI = Body mass index; N = Number of patients; IMC = Intermediate care; LOS = Length of stay.

### VSS Measurements and MET activation

**Fig 2:**
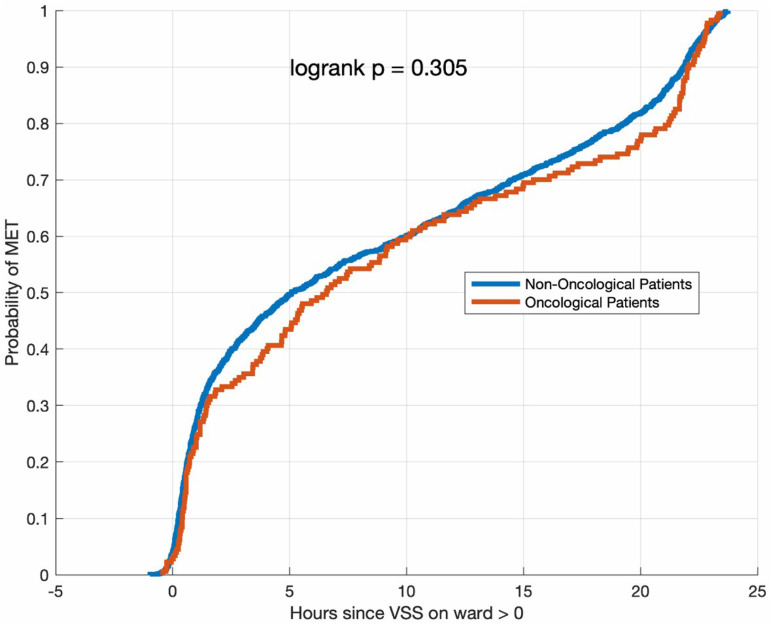
Log-rank test for the probability of MET after the first abnormal VSS.

Of the total 3,214 patients, 1,604 (49.9%) met the MET criteria in the 24 hours prior to MET activation (at least one time point with abnormal VSS prior to the assessment triggering the MET call). Among oncological patients, 177 out of 286 (61.9%) had an abnormal VSS prior to the MET activation, compared to 1,427 out of 2,928 (48.7%) of non-oncological patients (p < 0.001). Oncological patients had significantly more VSS measurements than non-oncological patients (median 24 [16–37] vs 18 [10–27], p < 0.001). This difference was became even more pronounced in patients with positive VSS scores (median 18 [10–27] vs 0 [0–3], p < 0.001, Supplementary Figure S1, S2 and S3 in S1 File). The most common reason for an abnormal VSS score was low systolic blood pressure (43.2%), followed by low oxygen saturation (34.1%). Supplementary Figure S4 in S1 File shows the distribution of pathological measurements for various vital signs in oncological and non-oncological patients. There was no significant difference in the time from the first abnormal VSS to MET activation between oncological and non-oncological patients (Fig 2).

### Primary outcome

In the analysis of the overall population, oncological status was significantly associated with 30-day mortality (OR 2.60, 95% CI 1.86–3.61, p < 0.001) after adjusting for age, admission type, and time from first abnormal VSS to MET activation (Table 2).

**Table 2:** Primary outcome 30-day mortality.

Variable	OR	95% CI Lower	95% CI Upper	p-value
First abnormal VSS to MET activation [per hour]	1.03	1.02	1.05	< 0.001
Additional estimated OR for first abnormal VSS to MET activation [per hour] in oncological patients (Interaction Term)	0.98	0.95	1.01	0.141
Age	1.03	1.02	1.04	< 0.001
Admission Type – Surgical*	0.58	0.48	0.70	< 0.001
Oncological patient	2.60	1.86	3.61	< 0.001

Legend: Logistic Regression Model for the Entire Population Including Interaction Term. OR = Odds ratio, CI = Confidence interval, VSS = Vital Sign Score. Admission Type: Medical or surgical.

**Fig 3:**
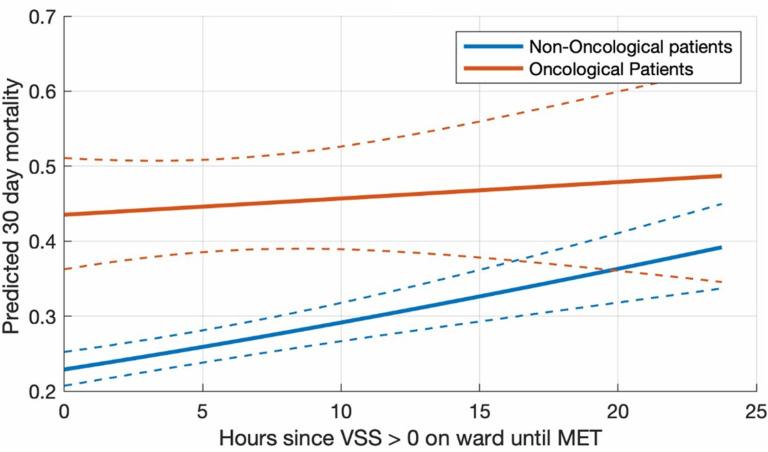
Logistic regression model for the two groups and the primary outcome 30- day mortality Legend: Predicted mortality in the total population as a function of hours since first abnormal VSS until MET activation, predicted for oncological and non-oncological patients according to the estimates provided in Table 2. The prediction assumes a median age of 69 years and a medical admission type.

Time from first abnormal VSS to MET activation was independently associated with increased 30-day mortality (OR 1.03 per hour, 95% CI 1.02–1.05, p < 0.001), with no additional increase in mortality risk in oncological patients (interaction term, p = 0.141), Fig 3.

### Subgroup of oncological patients

Time from first abnormal VSS to MET activation was not significantly associated with mortality in the oncological patient group (OR 1.02 per hour, 95% CI 0.99–1.06, p = 0.230) when the interaction term was included (p = 0.140). Among the oncological patients, the 30-day mortality rate was similar between haematological and solid tumour patients (36.4% vs 43.9%) (Supplementary Table S3 in S1 File). In the logistic regression model for oncological patients, tumour type (solid vs. haematological) did not significantly predict 30-day mortality (OR 1.68, 95% CI 0.84–3.39, p = 0.145). Age (OR 1.02 per year, 95% CI 1.01–1.00, p = 0.020) was a significant predictor of mortality in this subgroup (Table 3). Subgroup analysis of ICU patients can be found in the supplemental file (Figure S4 and Table S3 in S1 File).

**Table 3:** 30-day mortality in the subgroup of oncological patient.

Variable	OR	95% CI Lower	95%CI Upper	p-value
First abnormal VSS to MET activation [per hour]	1.02	0.99	1.06	0.230
First abnormal VSS to MET activation * tumour type	0.95	0.89	1.02	0.140
Age	1.02	1.00	1.04	< 0.001
Tumour type (solid)	1.68	0.84	3.39	0.145

VSS = Vital Sign Score

### Model performance

The predictive performance of the logistic regression models was assessed using the Area under the Receiver Operating Characteristic Curve (AUC-ROC). For the entire population, the model achieved an AUC-ROC of 0.67 (95% CI 0.64–0.68), a Nagelkerke’s pseudo R² of 0.091, and demonstrated good calibration (Hosmer-Lemeshow χ² = 4.70, p = 0.790). The ICU population model performed with an AUC-ROC of 0.71 (95% CI 0.67–0.74), a higher explained variance (Nagelkerke’s R² = 0.245), and adequate calibration (Hosmer-Lemeshow χ² = 12.80, p = 0.119). The oncological patient subgroup model had an AUC-ROC of 0.60 (95% CI 0.53–0.66), a Nagelkerke’s R² of 0.044, and good calibration (Hosmer-Lemeshow χ² = 7.00, p = 0.537).

## Discussion

This study highlights the impact of oncological status on patient outcomes and MET activations in a tertiary care setting. Approximately 10% of MET calls were for patients in the oncology ward. In our cohort, each hour of delay in MET activation was associated with a 3.2% increase in the odds of 30-day all-cause mortality. The absence of statistical significance for the interaction between oncological status and time to MET activation suggests a similar effect of delayed MET activation on mortality in both oncological and non-oncological patients [19]. However, given the higher baseline mortality risk in oncological patients and the significantly higher number of vital signs measurements, delays in critical care could have clinically meaningful consequences in this population [13]. The increased frequency of VSS measurements likely reflects greater awareness of these patients’ vulnerability or a lower threshold for initiating closer monitoring. However, despite this vigilance, the higher mortality rates suggest that early recognition of deterioration remains challenging. As Na et al. propose, integrating automated alert systems could improve timely responses to patient deterioration [20].

The discrepancy between increased VSS measurements and higher mortality rates in oncological patients raises important questions. It is possible that while these patients are monitored more closely, the interpretation of changes in their vital signs may be more complex due to the effects of their underlying disease or treatments. In addition, there might still be a reluctance to admit oncological patients to the ICU, which might be translated to the referring team [21]. Alternatively, the current VSS thresholds may not be optimally calibrated for oncological patients, potentially leading to delays in recognising deterioration. There may be a need for further research into tailored monitoring strategies and with potential adjusted VSS thresholds for oncological patients [5]. In addition, dedicated management algorithms, such as the MET management algorithm for chimeric antigen receptor (CAR) – T-cell recipients, might help improve the outcome of those patients [4].

The most common triggers for MET activation were concern, low oxygen saturation, and abnormal systolic blood pressure in oncological and non-oncological patients, with similar patterns observed in both groups. This consistency suggests that despite the differences in VSS measurement frequency, the criteria for MET activation remain similar across patient populations. Our analysis reveals that “concern” was a significant MET activation criterion, yet only one-third of these patients had abnormal VSS measurements. This suggests that VSS thresholds may not capture the full spectrum of clinical deterioration, particularly subtle changes that experienced clinicians recognize. Patients activated by “concern” had lower ICU admission rates, indicating they needed evaluation but often not critical care. More comprehensive early warning systems, such as NEWS, might better detect nuanced physiological changes, particularly in oncological patients whose deterioration patterns may differ [22]. Future research should compare different early warning systems and explore tailored thresholds for specific patient populations.

In our study, oncological patients exhibited significantly higher mortality rates compared to non-oncological patients, with a 30-day mortality rate of 38.1% versus 18.0%. This increased mortality risk persisted even after adjusting for disease severity using the APACHE II score in the ICU population, where oncological patients had 96% higher odds of 30-day mortality (Supplementary Table S1 in S1 File). However, in this patient population, the association of delayed MET intervention and mortality was less pronounced. Possibly, patients left on the ward were patients with “do not resuscitate” (DNR) orders in place or the MET intervention lead to a limitation of care. From the data we collected, we are unable to conclude whether the delay in MET activation caused an ICU admission to become obsolete.

In our population, oncological patients had longer hospital stays and those admitted to ICU had a higher APACHE II score, indicating the complexity of their care requirements. These findings are in line with other studies showing a high mortality in oncological patients. Mokard and colleagues found a mortality of 45.3% in neutropenic patients admitted to the ICU and identified the need for mechanical ventilation, bloodstream infection, renal replacement therapy and allogeneic transplantation as relevant risk factors [23]. Schellengowski and colleagues described a mortality of 55.0% in de novo acute myeloid leukaemia patients requiring intensive care [7]. Furthermore, in a previous analysis we could show, that chimeric antigen T-cell recipients who required MET intervention had a significantly higher 90-day mortality than those who did not (53% vs 1%) [11].

Our analysis revealed no significant difference in 30-day mortality between solid tumours and haematological malignancies. This is in contrast to data from Soares and colleagues, where solid metastatic and haematological cancer were associated with increased mortality [24], as well as findings by Na and colleagues, where critically ill patients with haematological malignancies had a higher mortality rate than patients with solid tumours [25]. However, in our study only patients referred from the medical oncology department were deemed oncological patients and patients with surgical treatment of their cancer were in the control group, which could have contributed to these results. In addition, MET activation for a positive VSS score or concern may select oncological patients at risk independent of their tumour type, suggesting that the type of cancer may be less important than overall oncological status in predicting short-term outcomes.

### Limitations

As our models demonstrated moderate discrimination ability as measured by AUC-ROC and show relatively low Nagelkerke’s R² values, additional factors such as specific treatments, comorbidities, or unassessed physiological parameters likely influence mortality outcomes and require further study. Nevertheless, the Hosmer-Lemeshow test results confirmed adequate model calibration across all populations, suggesting reliable risk estimation across different patient risk categories. As there is no standardized severity of illness scoring available for our non-ICU population, further statistical adjustment for severity of illness was not feasible. This represents a significant limitation, as oncological patients may have been more severely ill compared to the rest of the population, as indicated by their higher mortality rates. Vital parameters up to 24 hours before MET calls were retrospectively extracted from electronic records, with frequent missing data for GCS and respiratory rate, potentially underestimating the number of abnormal VSS measurements. Furthermore, we limited the extraction of vital signs (VSS) to the 24-hour period preceding MET activation, as we consider this timeframe to be the most clinically relevant in the context of delayed MET activation. If a patient exhibited abnormal VSS but did not trigger a MET activation for more than 24 hours, it is possible that the patient’s condition was less acute and did not necessitate immediate intervention. Consequently, our analysis may not fully capture the impact of earlier physiological derangements on outcomes and is applicable only to measurements within the 24-hour period prior to MET activation. In addition, as this is single-centre data, outcome comparisons across multiple ICUs are not feasible.

## Conclusion

Although outcomes did not differ significantly between oncological and non-oncological patients in our cohort, oncological patients inherently have a higher baseline mortality risk and represented approximately 10% of the study population. Notably, they underwent more frequent vital sign monitoring, suggesting increased clinical concern. Given these factors, we propose that oncological patients may benefit from a tailored approach, including earlier and more proactive Medical Emergency Team (MET) involvement. Delays in MET activation were associated with increased mortality across all patient groups, irrespective of oncological status. Therefore, individualized monitoring strategies, incorporating early MET review, may be particularly beneficial for this high-risk subgroup.

## Supporting information

S1 File Supplemental Digital Content. (DOCX)

## ABBREVIATIONS

APACHE II: Acute Physiologic Assessment and Chronic Health Evaluation
AUC-ROC: Area under the Receiver Operating Characteristic Curve
BMI: Body Mass Index
CI: Confidence Interval
DNR: Do not resuscitate
EC: Ethical Committee
GCS: Glasgow Coma Scale
ICU: Intensive Care Unit
IMC: Intermediate Care Unit
MET: Medical Emergency Team
OR: Odds Ratio
STROBE: STrengthening the Reporting of OBservational studies in Epidemiology
TTM: Time to MET activation
VSS: Vital Sign Score.
