# Supplementary material for: The critical role of timely medical emergency team activation in oncological and non-oncological patients
Source: PLoS One. 2025 Jun 2;20(5):e0324831. doi: 10.1371/journal.pone.0324831 (PMC12129341; doi:10.1371/journal.pone.0324831)
Supplement: S1 File — (DOCX) [file pone.0324831.s001.docx]

Supplemental Digital Content

This appendix has been provided by the authors to provide additional information

Supplement to: Bachmann KF, Michimura SJ, Cioccari L, Schefold JC, Messmer AS*.

**The critical role of timely medical emergency team activation in oncological and non-oncological patients**

*Corresponding author

Anna Messmer, MD

Department of Intensive Care,

Inselspital, Bern University Hospital and University of Bern

Freiburgstrasse 10

3010 Bern

Switzerland

ORCID: 0000-0002-3206-9112

**Appendix**

The critical role of timely medical emergency team activation in oncological and non-oncological patients

**Table of Contents Page**

[Subgroup analysis of patients admitted to the ICU 4](#_Toc194155463)

[Table S1. Baseline characteristics of oncological patients 5](#_Toc194155464)

[Table S2. Oncological diagnoses of patients requiring MET activation 6](#_Toc194155465)

[Table S3. 30-day mortality in the subgroup of Patients admitted to the ICU 7](#_Toc194155466)

[Figure S1. Number of VSS measurements 7](#_Toc194155467)

[Figure S2. Distribution of total VSS measurements 9](#_Toc194155468)

[Figure S3. Comparison of total VSS measurements 10](#_Toc194155469)

[Figure S4. Abnormal vital signs 14](#_Toc194155470)

[Figure S5. Logistic regression model for the two groups and the primary outcome mortality 16](#_Toc194155471)

# Subgroup analysis of patients admitted to the ICU

In the ICU subgroup analysis (Supplementary Table S1), including 1,457 patients, oncological status remained a significant predictor of 30-day mortality (OR 1.96, 95% CI 1.17-3.30, p = 0.011). The APACHE II score was also strongly associated with mortality (OR 1.08 per point, 95% CI 1.06-1.09, p < 0.001). TTM showed a borderline significant association with mortality in this subgroup (OR 1.02 per hour, 95% CI 1.00-1.03, p = 0.068), see Figure 3B. Again, there were no differences between the two groups' impact of delayed MET activation on mortality (interaction term, p = 0.697).

# Table S1. Baseline characteristics of oncological patients

| **Variable** | **Patients with solid tumor**  **N = 66** | **Patients with haematological tumor**  **N = 220** | **p-value** |
| --- | --- | --- | --- |
| Age, median years [IQR] | 65.0 [56.0-73.0] | 61.0 [49.0-68.0] | 0.011 |
| Sex, N (%)   - Male - Female | 52 (78.8)  14 (21.2) | 134 (60.9)  86 (39.1) | 0.009 |
| BMI (kg/m2), median [IQR] | 24.0 [21.3-26.6] | 24.8 [21.7-28.5] | 0.063 |
| APACHEII, median [IQR] | 18.5 [13.5-22.5] | 27.0 [22.0-32.5] | p < 0.001 |
| Admission Type, N (%)   - Surgical - Medical | 0 (0.0)  66 (100.0) | 0 (0.0)  220 (100.0) |  |
| MET calling criterion, N (%)   - Systolic blood pressure - Drop in GCS - Concern - Low oxygen Saturation - High or low heart rate - Threatened airway - Seizure - High or low respiratory rate | 9 (18.0)  2 (4.0)  17 (34.0)  8 (16.0)  8 (16.0)  5 (10.0)  0 (0.0)  1 (2.0) | 45 (25.0)  18 (10.0)  64 (35.6)  20 (11.1)  21 (11.7)  5 (2.8)  3 (1.7)  4 (2.2) | p = 0.368 |
| MET diagnosis group, N (%)   - Infectious - Respiratory - Neurological - Infectious, neurological - Cardiovascular - Psychiatric - Renal - Shock - *haemorrhagic* - Shock - *cardiovascular* - Metabolic, electrolyte disturbance - Gastrointestinal - Haematological - oncological - Other | 11 (16.7)  19 (28.8)  5 (7.6)  -  6 (9.1)  1 (1.5)  1 (1.5)  1 (1.5)  -  2 (3.0)  4 (6.1)  2 (3.0)  7 (10.6)  7 (10.6) | 87 (39.5)  43 (19.5)  19 (8.6)  2 (0.9)  20 (9.1)  2 (1.0)  3 (1.4)  4 (1.8)  1 (0.5)  0 (0.0)  7 (3.2)  12 (5.5)  5 (2.3)  15 (6.8) | 0.140 |
| Disposition, N (%)   - intensive care unit - ward - intermediate care unit | 22 (33.3)  42 (63.6)  2 (3.0) | 99 (45.0)  117 (53.2)  4 (1.8) | 0.230 |
| Hospital, median days [IQR] | 13.0 [4.0-25.0] | 27.0 [16.0-38.0] | 0.054 |

P-values were calculated using logistic regression, with the variable of interest as predictor and oncological status as the predicted variable.

# Table S2. Oncological diagnoses of patients requiring MET activation

| **Oncological Diagnosis** | **Number of Patients** | **Percentage (%)** |
| --- | --- | --- |
| Acute Myeloid Leukemia (AML) | 65 | 22.73 |
| B-Cell Lymphoma | 56 | 19.58 |
| Multiple Myeloma | 36 | 12.59 |
| Haematologic Malignancy (other) | 28 | 9.79 |
| Malignant neoplasms of the lung | 19 | 6.64 |
| Acute Lymphoblastic Leukemia (ALL) | 16 | 5.59 |
| T-Cell Lymphoma | 9 | 3.15 |
| Malignant neoplasms of the mouth | 8 | 2.8 |
| Malignant neoplasms of the oropharynx | 8 | 2.8 |
| Hodgkin Lymphoma | 8 | 2.8 |
| Malignant neoplasms of the stomach | 5 | 1.75 |
| Malignant neoplasm of testis | 5 | 1.75 |
| Malignant neoplasm of the intestines | 4 | 1.4 |
| Malignant neoplasms of the oesophagus | 4 | 1.4 |
| Malignant neoplasm of the urogenital tract | 4 | 1.4 |
| Chronic Lymphocytic Leukemia (CLL) | 2 | 0.7 |
| Malignant neoplasm of the breast | 2 | 0.7 |
| Malignant melanoma | 2 | 0.7 |
| Malignant neoplasms of the vulva | 1 | 0.35 |
| Malignant neoplasm of brain | 1 | 0.35 |
| Malignant neoplasms of the uterus | 1 | 0.35 |
| Malignant neoplasms of peripheral nerves | 1 | 0.35 |

# Table S3. 30-day mortality in the subgroup of Patients admitted to the ICU

| **Variable** | **OR** | **95% CI Lower** | **95% CI Upper** | **p-value** |
| --- | --- | --- | --- | --- |
| First abnormal VSS to MET activation [per hour] | 1.02 | 1.00 | 1.03 | 0.068 |
| Additional estimated OR for first abnormal VSS to MET activation [per hour] in oncological patients (Interaction Term) | 0.99 | 0.94 | 1.04 | 0.697 |
| APACHEII (per point) | 1.08 | 1.06 | 1.09 | < 0.001 |
| Oncological patient | 1.96 | 1.17 | 3.30 | 0.011 |

Logistic regression model for the ICU population including interaction term. VSS = Vital Sign Score.

Figure S1. Number of VSS measurements


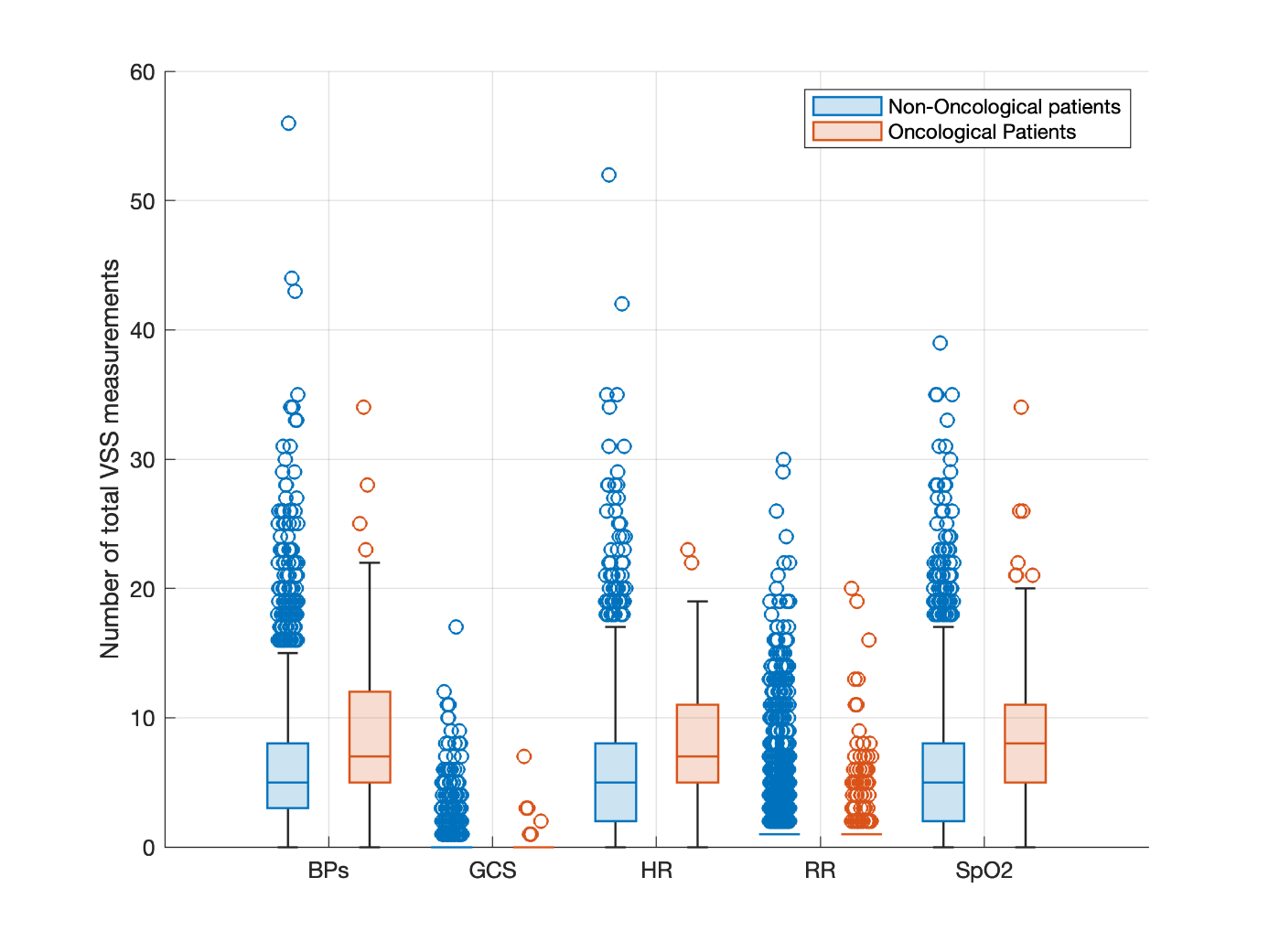


The number of VSS measurements of non-oncological and oncological patients on the ward, grouped according to the vital sign that was measured. BPs: Systolic Blood Pressure. GCS: Glascow Coma Scale. HR: Heart Rate. RR: Respiratory Rate. SpO2: Oxygen Saturation

# Figure S2. Distribution of total VSS measurements


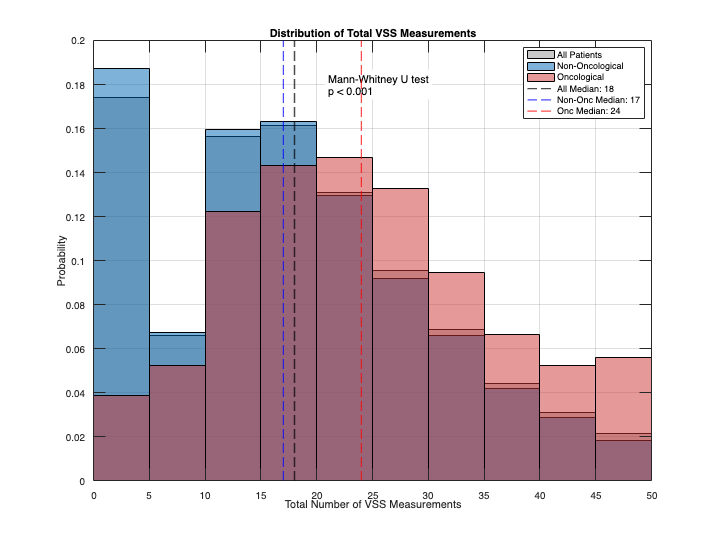


# Figure S3. Comparison of total VSS measurements


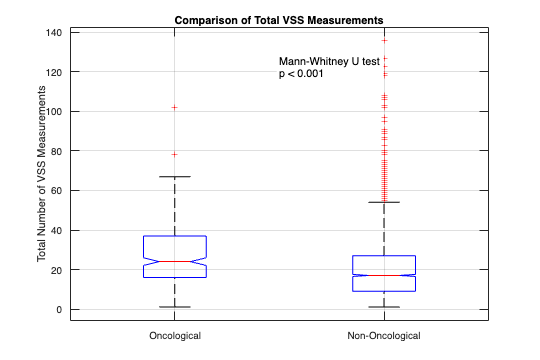


# Figure S4. Abnormal vital signs


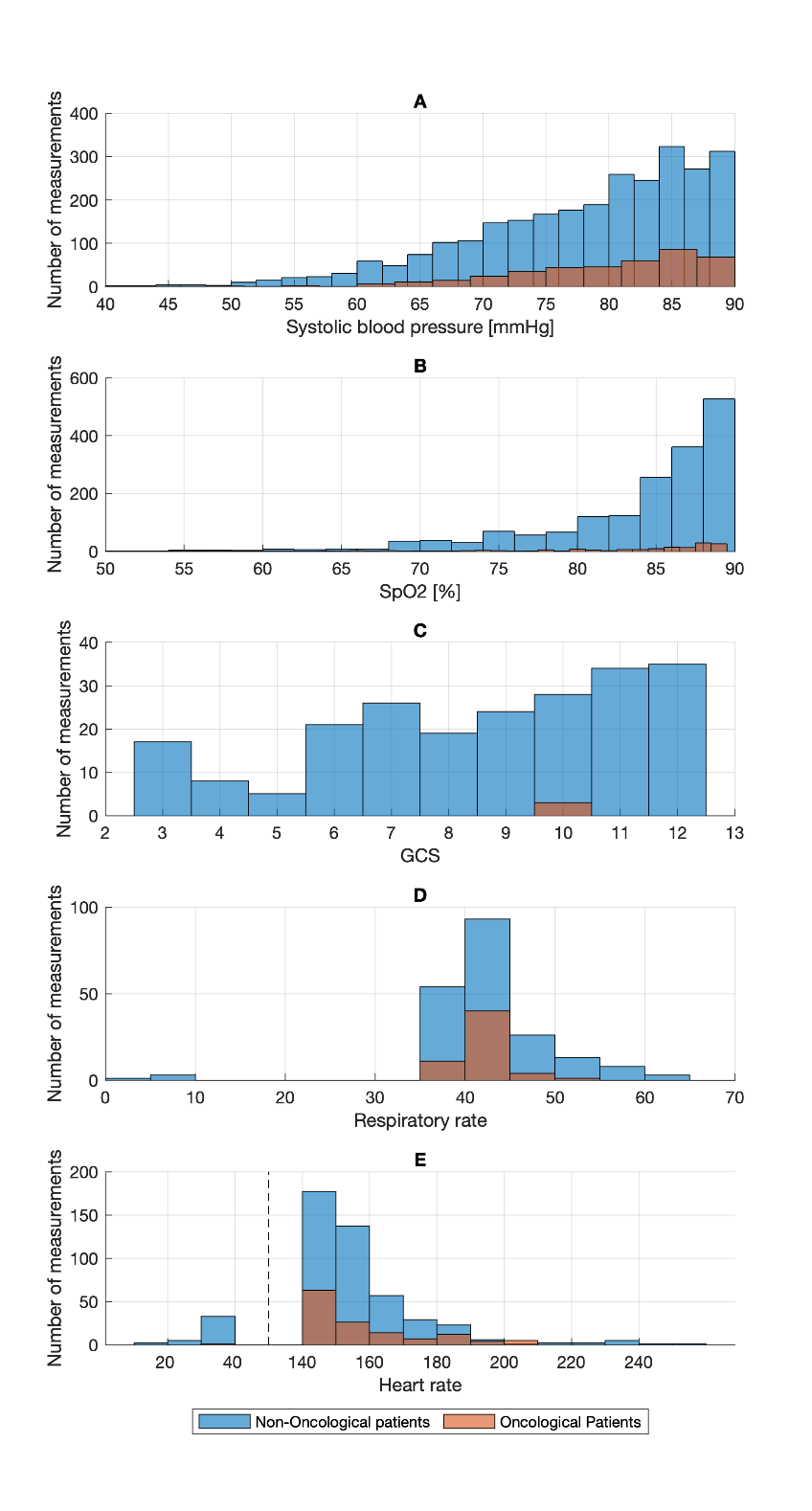


Abnormal measurements according to the vital sign score (VSS), grouped into non-oncological (orange) and oncological (blue) patients.

# Figure S5. Logistic regression model for the two groups and the primary outcome mortality


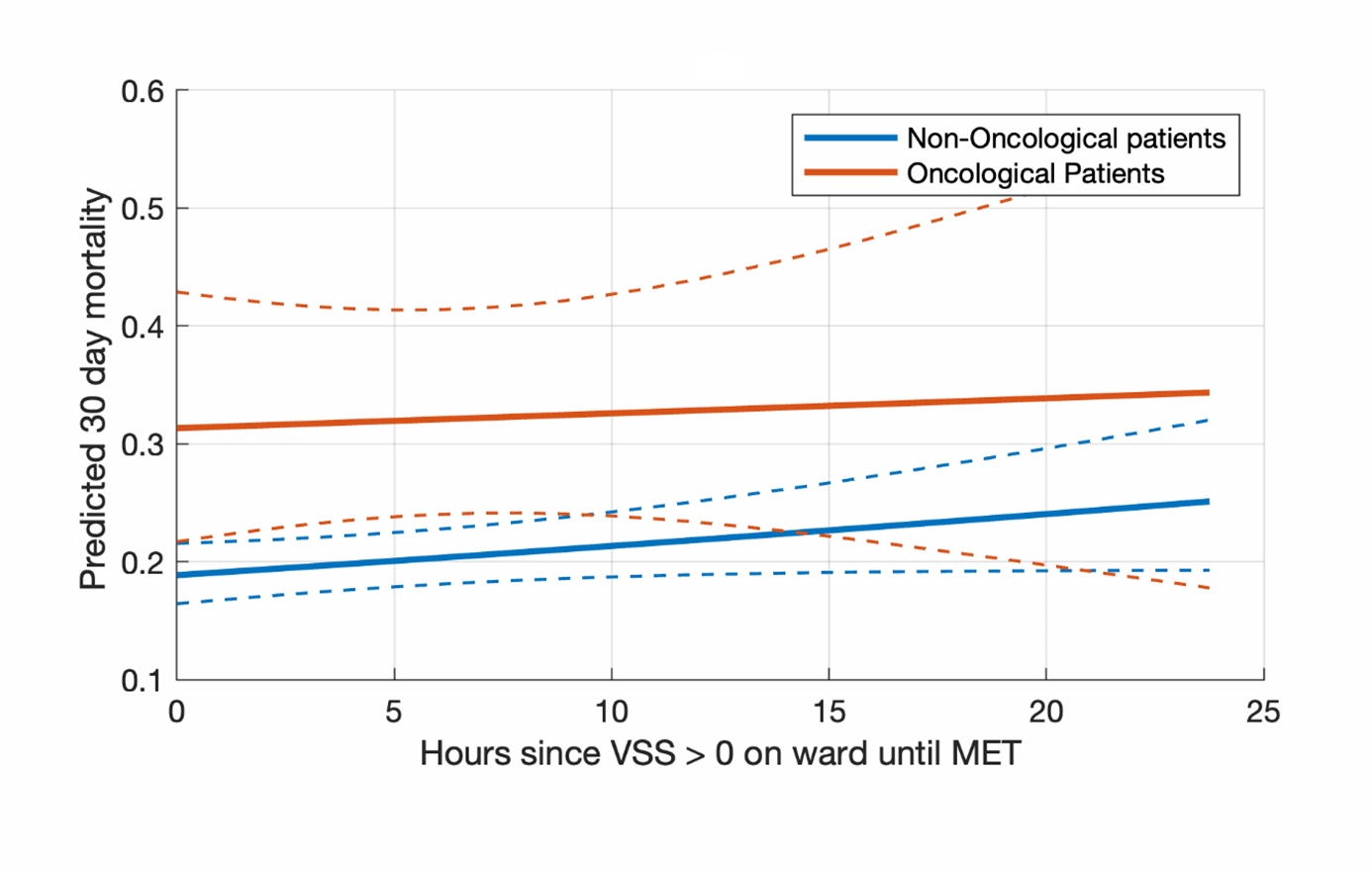


Predicted mortality in the ICU population as a function of hours since VSS > 0 until MET, predicted for oncological and non-oncological patients according to the estimates provided in Table S1. The prediction assumes a median APACHEII score of 22.
